# Development and Characterization of a Preclinical Model of Breast Cancer Lung Micrometastatic to Macrometastatic Progression

**DOI:** 10.1371/journal.pone.0098624

**Published:** 2014-05-30

**Authors:** Lora C. Bailey-Downs, Jessica E. Thorpe, Bryan C. Disch, Anja Bastian, Paul J. Hauser, Taleah Farasyn, William L. Berry, Robert E. Hurst, Michael A. Ihnat

**Affiliations:** 1 DormaTarg, Inc., Oklahoma City, Oklahoma, United States of America; 2 Department of Pharmaceutical Sciences, University of Oklahoma College of Pharmacy, Oklahoma City, Oklahoma, United States of America; 3 Department of Urology, University of Oklahoma College of Medicine, Oklahoma City, Oklahoma, United States of America; 4 Department of Cell Biology, University of Oklahoma College of Medicine, Oklahoma City, Oklahoma, United States of America; 5 Department of Biochemistry and Molecular Biology, of Oklahoma College of Medicine, Oklahoma City, Oklahoma, United States of America; University of L’Aquila, Italy

## Abstract

Most cancer patients die with metastatic disease, thus, good models that recapitulate the natural process of metastasis including a dormancy period with micrometastatic cells would be beneficial in developing treatment strategies. Herein we report a model of natural metastasis that balances time to complete experiments with a reasonable dormancy period, which can be used to better study metastatic progression. The basis for the model is a 4T1 triple negative syngeneic breast cancer model without resection of the primary tumor. A cell titration from 500 to 15,000 GFP tagged 4T1 cells implanted into fat pad number four of immune proficient eight week female BALB/cJ mice optimized speed of the model while possessing metastatic processes including dormancy and beginning of reactivation. The frequency of primary tumors was less than 50% in animals implanted with 500–1500 cells. Although implantation with over 10,000 cells resulted in 100% primary tumor development, the tumors and macrometastases formed were highly aggressive, lacked dormancy, and offered no opportunity for treatment. Implantation of 7,500 cells resulted in >90% tumor take by 10 days; in 30–60 micrometastases in the lung (with many animals also having 2–30 brain micrometastases) two weeks post-implantation, with the first small macrometastases present at five weeks; many animals displaying macrometastases at five weeks and animals becoming moribund by six weeks post-implantation. Using the optimum of 7,500 cells the efficacy of a chemotherapeutic agent for breast cancer, doxorubicin, given at its maximal tolerated dose (MTD; 1 mg/kg weekly) was tested for an effect on metastasis. Doxorubicin treatment significantly reduced primary tumor growth and lung micrometastases but the number of macrometastases at experiment end was not significantly affected. This model should prove useful for development of drugs to target metastasis and to study the biology of metastasis.

## Introduction

Most cancer deaths are due to metastasis to distal sites or recurrence at the site of the primary tumor [1]. In spite of this long-known fact, most major anticancer therapy - surgery, radiation and chemotherapy – is much better at treating the primary tumor [2], [3]. In fact, many metastasis animal models are not designed to study micrometastases, key intermediates in the metastatic process. Micrometastases are enticing therapeutically because they begin as single cells and can exist as such or as small clumps of cells for long periods of time, thus at least in theory overcoming tumor heterogeneity [4]. It has also been shown that metastases accumulate genetic, epigenetic and proteomic changes and a resistance to treatment not present in the primary tumor [5]–[7]. Thus attacking micrometastases before they become metastases could - again in theory - represent a more viable therapeutic target. One key drawback in examining micrometastases is that no natural model to study this process has yet been developed. Tumor implantation models of metastasis typically use large numbers of cells, removal of the primary tumor and oftentimes use animals lacking key immune cells to drive the process to macrometastasis in a small length of time [8]. A few genetic tumorigenesis models have been shown to form metastases; but these often take several months to years to develop, use one or two key mutations to drive tumorigenesis, and are very expensive [9]–[12].

When developing this metastatic progression model, some key desired characteristics were: 1) that the tumor represents a clinically relevant type; 2) that tumor implantation and primary tumor measurement be technically reasonable, so that the model could be used with only brief training and with equipment available in most laboratories; 3) that primary tumor and micrometastasis take be reproducibly high so that few animals are wasted; 4) that the animals have an intact immune system, since this is an emerging hallmark of cancer, metastasis in particular [13]; 5) that micrometastases activate to form macrometastases over time, such that the entire process can be studied and that there be a window of time where just micrometastases are present, so that these can be better studied and hopefully targeted; and 6) that the cellular model be annotated in literature, so that basic characteristics and tools to study the model are available. The 4T1 mouse breast model met all these criteria being that it is 1) a clinically relevant triple negative breast cancer metastatic to the lungs, brain and bone [14]; 2) implantation into the mammary fat pad and caliper measurement of primary tumors is technically easy; 3) as shown below, after optimizing cell number, primary tumor and lung micrometastasis take is almost quantitative; 4) the tumor cells being mouse in origin, immune proficient, inexpensive BALB/cJ mice are used as the host; 5) that, as shown below, lung micrometastases are observed soon after implantation, exist alone for a few weeks then activate to become macrometastases; and 6) the 4T1 model is well described in the literature, with over 800 citations on the model as of the time of this publication.

In this work we sought to modify the 4T1 model to study the continuum of lung micrometastasis to macrometastasis. We report that implantation of 7,500 4T1 cells into a lateral mammary fat pad is optimal for primary tumor and micrometastasis take; gives a three-four week window when only micrometastases are present, and by five weeks post implantation forms lung macrometastasis.

## Methods

### Cell Culture

4T1 Luc2GFP cells (PerkinElmer, Waltham, MA) were cultured in DMEM high glucose media with 2 mM pyruvate, 2 mM glutamine, 2% Pen/Strep and 10% Cosmic Calf Serum (Mediatech, Manassas, VA). Cells were allowed to grow at 37°C with 95% humidity for one-two days before use in animals. To prepare cells for injection implantation, 4T1 Luc2GFP cells were trypsinized, removed from flasks, verified to be in single cell suspension, cells counts and viability were then accessed by the TC10 counting system (BioRad, Hercules, CA). Briefly, 50 µL of trypan blue was added to 50 µL cell suspension and mixed by pipetting gently; 10 µL of the trypan blue-cell suspension mixture was then loaded into the TC10 counting chamber. The chamber was then placed into the TC10 counter, which is an automated cell counter that provides both cell number and viability by trypan blue exclusion. The remaining cells were then washed with phosphate buffered saline (PBS), pelleted at a concentration of 75,000 cells/mL and suspended in 1 mL of fresh PBS containing 1 mM EDTA. Once suspended, cells remained at room temperature and were injected into the mouse within 20 minutes. Proper handling and quick implantation proved to be crucial to obtaining consistent and reproducible tumor take. For instance, after only 10 minutes on ice, cell viability was substantially reduced. Additionally, if cells were not implanted within twenty minutes of trypsinization, tumor take was reduced. This was determined simply by noting the time of trypsinization and implantation. The animals implanted with cells >20 minutes after trypsinization had decreased tumor take (observations from every experiment) and thus these cells were not used for implantation.

### Primary Tumor Implantation

A 1cc syringe without needle was used to draw 600 µL (enough for five animals) of cell suspension from a microcentrifuge tube with 4T1 Luc2GFP cell suspension (prepared as above described). A 25-gauge needle was then placed onto the syringe and air was carefully but thoroughly removed. 100 µL of cell suspension was injected subcutaneously (bevel side up) into mammary fatpad number four of eight week old female BALB/cJ mice (NCI National Laboratory Frederick/Charles River animal production program). To aid in the correct placement of the injection, the fatpad was identified, marked, and the surrounding area was shaved the day before implantation. Since the animals were injected without sedation, a second person was required to properly restrain the mice. The animals were grasped behind the neck firmly using the thumb and index finger, its body stretched along the hand, and its tail and feet were restrained using the last two fingers. The animals were held in a manner that pulled the skin of the abdomen taut and therefore allowed for easier implantation of cells. All attempts were made to minimize animal distress, which was assessed by weekly weight measurements and the presence of physiological signs of distress (piloerection, vocalizations, recumbent posture, or changes in eating habits). Animals were housed in the Comparative Medicine animal facilities at OUHSC College of Pharmacy. All procedures were in keeping with American Association for Laboratory Animal Science (AALAS) standards and were approved by the Institutional Animal Care and Use Committee (IACUC) at OUHSC in accordance to protocol number 11-038-H.

### Cell Titration

BALB/cJ female mice were implanted with 500, 750, 1000, 1,500, 7,500, or 15,000 4T1-Luc2GFP cells. All animals were assessed for tumor take (tumor take was assessed from no fewer than nine independent experiments). Six weeks after implantation, the animals were humanely euthanized by overexposure to carbon dioxide (according to AAALAS guidelines). Micrometastasis and macrometastasis were determined and quantified at experimental end from no fewer than 15 animals per group in two independent experiments.

### Time Course

BALB/cJ female mice (two independent experiments containing an n of 5–8 per group and 7–11 per group) were implanted with 7,500 4T1-Luc2GFP cells. All animals were weighed and tumors were measured using calipers three times weekly. One group of animals per week for five weeks were isoflurane-sedated and retro-orbitally injected with 100 µL of tetramethylrhodamine (TMR)-labeled 2×10^6^ MW dextran (Invitrogen Molecular Probes; Eugene, Oregon) at a concentration of 2.5 mg/mL. The injection of TMR-labeled dextran allows for the visualization and assessment of vascularity in tumors and lungs. The animals were allowed to recover for fifteen minutes after injection and were then humanely euthanized by overexposure to carbon dioxide (according to AAALAS guidelines).

### Bone Metastasis in 4T1-TR (Tomato Red) Model

BALB/cJ female mice (*n* = 20) were implanted with 7,500 4T1 cells labeled with dtTomato (kind gift from Ralf Janknecht lab). All animals were weighed and tumors were measured using calipers three times weekly. After five weeks, the animals were humanely euthanized by overexposure to carbon dioxide (according to AALAS guidelines). The lung, brain, spleen, liver, femur, patella, tibia, and fibula were all harvested at time of euthanization for assessment of metastasis. TR positive cells were imaged under examination with excitation of TR emission using a Leica Model Z16 APO fluorescence microscope equipped for a wide field, a large depth of field and a 10× zoom capability.

### Doxorubicin Efficacy in 4T1-Luc2GFP Model

BALB/cJ female mice (two independent experiments with an *n* = 10 per group and *n* = 15 per group) were implanted with 7,500 4T1-Luc2GFP cells. Based on previous work in our laboratory, an maximal tolerated dose (MTD) of 1 mg/kg of doxorubicin in carrier (5% Pharmasolve and 5% Solutol HS in normal saline) was given by intraperitoneal administration once weekly [15]. All animals were weighed and tumors were measured using calipers three times weekly. Five weeks after implantation all animals were isoflurane-sedated and retro-orbitally injected with 100 µl of tetramethylrhodamine (TMR)-labeled 2×10^6^ MW dextran (Invitrogen Molecular Probes; Eugene, Oregon) at a concentration of 2.5 mg/mL. The animals were allowed to recover for 15 minutes after injection and were then humanely euthanized by overexposure to carbon dioxide (according to AAALAS guidelines).

### Primary Tumor Removal and Imaging

For all above experiments, the primary tumor was carefully dissected away from surrounding tissues, weighed, imaged, and prepared for paraffin embedding. All tumors were imaged using Leica Model Z16 APO fluorescence dissecting microscope equipped for a wide field, a large depth of field and a 10× zoom capability. The image settings used for primary tumor visualization were 0.57× with 1× objective exposed for 208 milliseconds using the GFP filter set. TMR-labeled dextran was visualized in the tumor using the FITC filter set with an exposure of 12.3 milliseconds with the magnification of 0.57× using the 1× objective lens. All tumors were imaged using the exact same image settings. The lungs, brain, liver, and spleen were then isolated, cleaned with phosphate buffered saline, imaged, and assessed for GFP positive cells (see Metastasis assessment).

### Tumor Preparation and Composition Determination

At the end of experiments, tumors were incubated in 4% buffered formaldehyde overnight. Tumors were then paraffin embedded and sectioned onto slides, which were then stained with hematoxylin and eosin. Histological examination of tumors was performed by an OUHSC pathologist to determine the composition of the tumors excised. It was confirmed that the tumors consisted of mammary gland acini and subcutaneous fat without excess stromal tissue. This assessment was performed to ensure proper implantation, tumor development, and reproducibility of the procedure.

### Metastasis Assessment

GFP and TR positive cells in freshly removed organs were imaged and quantified after excitation of GFP or TR using a Leica Model Z16 APO fluorescence dissecting microscope equipped for a wide field, a large depth of field and a 10× zoom capability. GFP positive objects were counted and scored based on macrometastases, large micrometastases, or small micrometastases in a blinded fashion by the microscopic operator. Overlay images taken at GFP and TMR excitation and emission wavelengths were used to assess whether a given cluster of cells had attracted a blood supply. Macrometastases were defined as being a cluster of at least 10 cells that has attracted a blood supply as indicated with TMR overlay. Large micrometastases were defined as a cluster of at least 10 cells with no evidence of a blood supply. Small micrometastases were defined as solitary micrometastatic cells or fewer than 10 cells with no evidence of angiogenesis. The assessment of GFP cells required the usage of multiple focal planes and magnification to confirm both the cell number and vascularity both of which were determined by the microscope operator. As expected, copious amounts of TMR-labeled dextran remained in the highly vascularized lung tissue, making it necessary to reduce the exposure for vascular assessment especially for micrometastases assessment. The authors highly recommend using the highest level of magnification possible to determine the presence of vessels in these metastatic tumors. Typically, analysis of lungs from a single animal took 35–50 minutes.

### Retrieval of 4T1-Luc2GFP Cells from Lung

After quantification and characterization of metastases in the lung, both lungs of the mouse were removed, washed with Hanks balanced salt solution (HBSS) (Corning-Costar, Corning, NY), minced into small pieces with scissors, and digested in 5 mL HBSS, containing 4 mg/mL collagenase type IV (Sigma-Aldrich, St Louis MO), 2.5 U/mL DNase I (Roche, Basel Switzerland), and 10 U/mL penicillin/streptomycin ([16] with modifications). Samples were placed in a hybridization incubator (Lab-Line, Melrose Park, IL) and rotated at 37°C for 30 minutes. Samples were then broken up by repeated pipetting, again rotated for 15 minutes at 37°C, followed by a second pipetting, and lastly filtered through a 40 µm cell strainer. HBSS (20 mL) was added and samples were centrifuged for five minutes at 700×*g*. Cell pellets were suspended in HBSS with 0.1% BSA, counted then analyzed for GFP positive cells by flow cytometry using a BD FACSCalibur (BD Scientific, Franklin Lakes, NJ).

### Statistical Analysis

Graph generation and statistical analysis were done on Prism 6.0 software (GraphPad, San Diego CA). Tumor growth rates of doxorubicin and carrier treated animals were calculated and groups compared using two-way ANOVA with Repeated Measures post-test and significance determined at p<0.05. Comparisons (one-way ANOVA with Neuman-Keuls, Tukey, or Bonferroni post-test; significance at p<0.05) were made among drug treated groups as compared to carrier treated groups with respect to metastasis.

## Results

**Figure 1:**
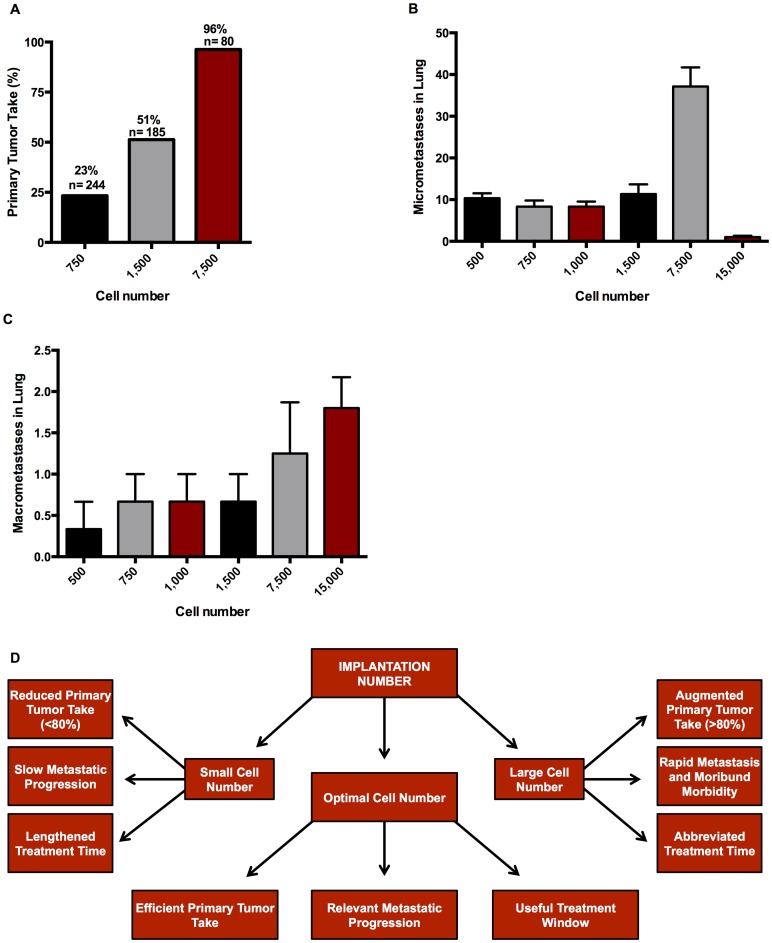
Summary of optimal cell number determination in the development of the metastatic mouse model. (**A**) Chart indicating the benefits of optimizing cell number for primary tumor take and metastatic progression in the 4T1 breast cancer model. (**B**) Percent tumor take in immune-proficient BALB/cJ mice implanted with 4T1-Luc2GFP cells into the number four mammary fat pad. Data are actual percent tumor take based on number of animals indicated above each bar from no fewer than nine independent experiments. Micrometastasis (**C**) and macrometastasis (**D**) in lung six weeks after implantation. Data are mean ± *SEM* from two independent experiments with *n* = 5 for experiment one, *n* = 10 for experiment two.

Most 4T1 metastatic breast cancer animal model papers implant at least 40,000 cells and remove the primary tumor before it gets large and impinges on animal health in order to push the system toward macrometastasis (**Table 1** and [14]). In an attempt to develop a model that recapitulates all the steps that occur naturally, we chose to start at lower numbers of cells without removing the primary tumor. To characterize the cell number dependent latency of tumor formation, a broad range cell titration from 500 to 15,000 4T1-Luc2GFP cells was performed. Tumors became palpable within 10 days in animals implanted with 7,500–15,000 cells and by 17 days in animals implanted with 500–1,500 cells. In a second series of titrations performed using 750, 1,500, and 7,500 cells, the percentage of animals that developed tumors at day 14 varied from 23% with 750 cells, 50% with 1500 cells, to over 90% with animals implanted with over 7500 cells (**Fig. 1B**). A comparison of lung metastases at six weeks in animals implanted with 500–7,500 cells had mostly lung micrometastases (**Fig. 1C**) with a few macrometastases (**Fig. 1D**) per lung at six weeks; while animals implanted with 15,000 cells had mostly larger metastases (**Fig. 1D**) with few micrometastases (**Fig. 1C**) Six weeks post implantation. Together, a flowchart behind the findings is shown in the diagram in **figure 1A**.

**Table 1:** Review of literature on the 4T1 model being used for metastasis evaluation.

PMIDand yearof paper	Number of cellsimplanted intomurine model	Evaluationtime (afterimplant)	Metastasis: Assessment typeand values (if applicable).
1540948 (1992) [35]	1×10^5^ 4T1 cellsimplanted 10 uL	Weekly	Qualitative assessment of murine metastases in blood (38/51 mice),draining lymph nodes (12/58), in lung (5/5 at day 14),and in liver (5/5 at day 28).
9537252 (1998) [36]	5×10^3^ 4T1 cells	Time course	Visual quantification of methylene blue stained metastatic cellsharvested from murine lung. Analysis at days: 14–18(13/13 mice with 1–43 cells); 22 (*n* = 6/11 with 32–338 cells);30–32 (*n* = 10/10 with 6–116,500 cells); 34–37(*n* = 10/12 with 315-267K cells); >42 (*n* = 14/14 with1–200K cells).
10411109 (1999) [37]	1×10^5^ 4T1 (clone)cells/mL implanted10 µL	34.0and 36.6 days	Visual quantification of metastases in India ink inflatedmurine lung. (*n* = 6, mean of 22.5+/−8.5) (*n* = 15,mean of 34.3+/−2.7).
12070302 (2002) [38]	0.5×10^5^ 4T1 cellsimplanted.	8 weeks	Primary tumors removed after 10 days. Visual quantificationof metastases in murine lung *n* = 5 mean of11.4+/−2.1 and liver *n* = 5 mean of 6.4+/−1.31.
12107848 (2002) [39]	5×10^4^ 4T1 cellsimplanted 100 µL	2 weeks	Visual quantification of metastatic nodules in murine lung.Identified 17 nodules per lung in all untreated animals (*n* = 6).
15210113 (2004) [40]	5×10^5^ 4T1 cellsimplanted 20 µL	Weekly	Visual quantification of metastatic nodules inmurine lung (*n* = 15–20 mean of 105+/−14).
15240548 (2004) [41]	1×10^6^ 4T1/Luc cellsimplanted 100 µL	3 weeks	Qualitative assessment of metastatic nodules in murinelung and bone by luciferase activity and H&Eanalysis. No quantification indicated (*n* = 10).
15161056 (2004) [17]	1×10^5^ 4T1 cells	4–5 weeks	Qualitative assessment of murine heart and lungmicrometastases and quantitative analysis oflarge lung metastases (*n* = 5–11).
**15574767 (2004) ** [42]	7×10^3^ 4T1-GFP-FL(firefly luciferase)cells	5–6 weeks	Primary tumor removed after 21 days. NoninvasiveBLI and MRI used for qualitative assessment ofliver, lung, brain, heart, kidney, spleen, bone,intestine, and subcutaneous metastases in livingmice. Metastases verified by *ex vivo* imaging ofluciferase activity or culture of harvestedtumor cells. (n = 8).
**15627887 (2004) ** [43]	7×10^3^ 4T1 cells	3 weeks	Qualitative visual assessment of metastasisfollowing india ink inflation of murine lung (*n* = 5).
**15854801 (2005) ** [44]	1×10^7^ 4T1 cellsimplanted 100 µL	∼4 weeks	Visual quantification of metastases on thesurface of the lung (*n* = 6 mean of 45+/−5).
**15978719 (2006) ** [45]	1×10^7^ 4T1 cells/mL,100 µL volumeimplanted	∼3 weeks (19 days)	Visual quantification of metastases on the surface ofmurine lung tissue fixed in diluted Bouin’s solution(*n* = unknown with 60 metastases per lung).
**16778210 (2006) ** [46]	4×10^4^ 4T1 cellsimplanted	4 weeks	Visual quantification of metastases in murine lungtissue stained with hematoxylin and eosin (*n* = 11–13with 50–100 nodules/lung.
**17132226 (2006) ** [47]	1×10^5^ 4T1cells/mL implanted100 µL	40 days	Visual quantification of pulmonary surfacemetastases (*n* = 20 untreated mice with 1to >20 metastases.
**17266169 (2007) ** [48]	1×10^5^ 4T1 cells	4 weeks	Visual quantification of metastases on the surfaceof murine lung tissue fixed in diluted Bouin’ssolution (*n* = 8–11 with 40–50 metastases per lung).
18691423 (2008) [49]	1×10^7^ cells/mL4T1/Luc implanted100 µL	6 weeks	Qualitative assessment of metastases in lung (6/6 mice),liver (5/6 mice), spleen (3/6 mice) and bone(2/6 mice). Metastases were occasionally foundin lymph nodes, brain, intestine, kidneys, and adrenals.
**19643025 (2009) ** [50]	1×10^6^ 4T1 cells	One week after tumor weight = 0.2 g	Visual qualitative assessment of murine pulmonarymetastasis (*n* = 10 with metastasis reported in alluntreated animals.
**19916050 (2010) ** [18]	1×10^4^ 4T1 cells	3–4 weeks	Qualitative assessment of micrometastases (startingday 23) and large metastases (starting day 28)in murine lung stained with hematoxylin andeosin (*n* = 3 mice per experimental groupwith approximately 50 metastases per mouse).
20351690 (2010) [51]	1×10^6^ 4T1 cellsimplanted25 µL	4 weeks	Visual quantification of metastases size in murinelung tissue stained with hematoxylinand eosin (*n* = 5).
21834963(2011) [52]	5×104 cells in0.1 mlMatrigelvolume	25 days	Visual quantification of metastases on thesurface of murine lung tissue fixed indiluted Bouin’s (*n* = 15).

**Figure 2:**
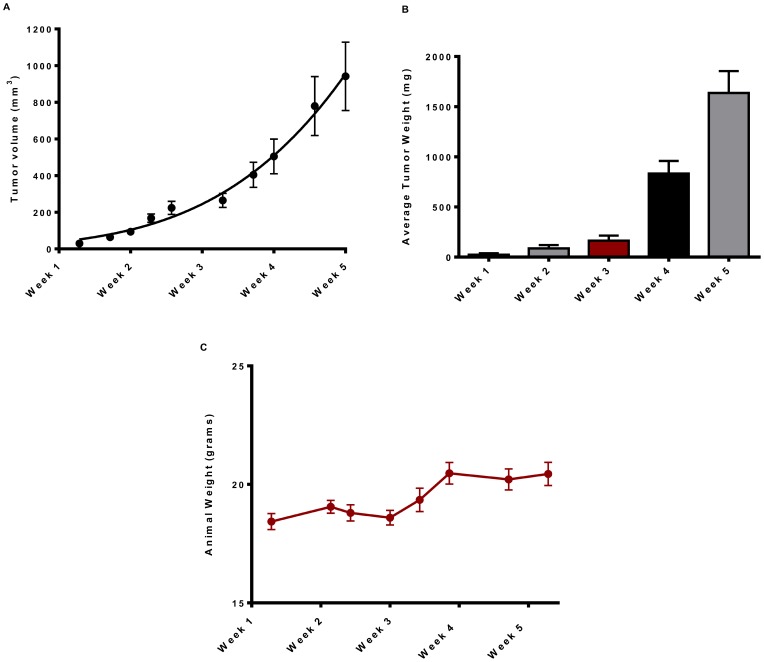
Growth of 4T1-Luc2GFP primary tumors: Timecourse. Primary tumor growth was recorded over a five week period from BALB/cJ mice implanted with 7,500 4T1-Luc2GFP cells into the mammary fat pad. (**A**) Tumor volume (mm^3^). (**B**) Tumor weight (mg). (**C**) Animal weights (g). Tumor volumes were calculated using the ellipsoidal method, volume (mm^3^) = 0.52×length × width^2^. All data are mean ± *SEM* from two independent experiments with 5–8 mice for experiment one and 7–11 mice for experiment two and are fitted to a Gomperzian growth curve by Prism 6.0 software.

With over 90% of animals having primary tumors present by day 10 in animals injected with 7,500 cells and mostly micrometastases with a few macrometastases at weeks five-six, it was decided that 7,500 cells produced an optimal model adequately representing all the steps of metastasis with cost and time constraints. Using this model, tumors grew in a Gompertzian fashion to an average volume of 1000 mm^3^ (**Fig. 2A**) and tumor weights grew to around 2 g (**Fig. 2B**) at five weeks post-implantation. Finally, animal weights remained steady and grew at later weeks after implantation (**Fig. 2C**).

**Figure 3:**
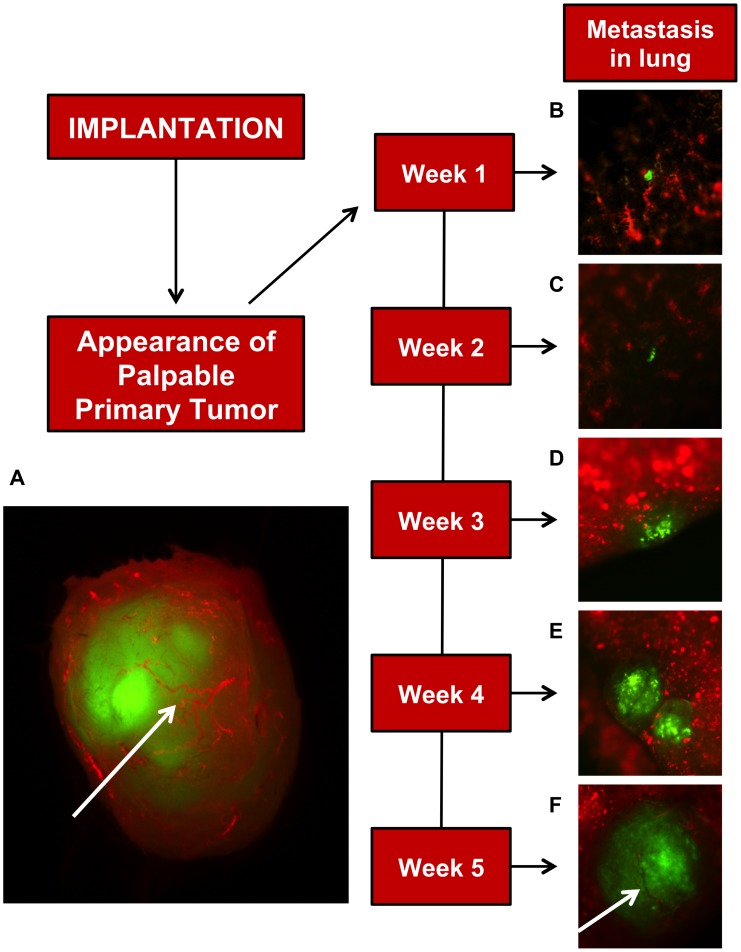
Development of lung metastasis in the 4T1 Luc2GFP mouse model: Timecourse. (**A**) Fluorescent image of a fully vascularized primary tumor removed five weeks after 7,500 4T1-Luc2GFP cells (green in all images) implanted into BALB/cJ mammary fat pad. Vasculature (red) in primary tumor (**A**) and five weeks lung metastasis (**F**) are indicated by arrows and was achieved by retro-orbital injection of tetramethylrhodamine labeled 2×10^6^ MW dextran. At weeks one and two (**B** and **C**) micrometastases, which are defined as single-to small clusters of cells, are present in lungs. Large micrometastases lacking blood vessels are present in lungs by weeks three and four (**D** and **E**), and by week five (**F**) macrometastases containing visible blood vessels are present in the lungs.

**Figure 3** shows representative images of a primary tumor and classification of metastatic tumors taken from this model. **Figure 3A** confirms the highly vascularized nature of the primary tumor. **Figure 3B** and **figure 3C** illustrate small micrometastases. Large micrometastases are shown in **figure 3D** and **figure 3E**. A macrometastasis is shown in **figure 3F**. It was also found that GFP-tagged cells from these macrometastases can be isolated using enzymatic digestion of lungs and FACS sorting of GFP positive cells. Using this method, around 600 cells can be isolated from a large lung macrometastasis taken at five weeks, giving adequate cell numbers for genetic and sensitive biochemical assays.

**Figure 4:**
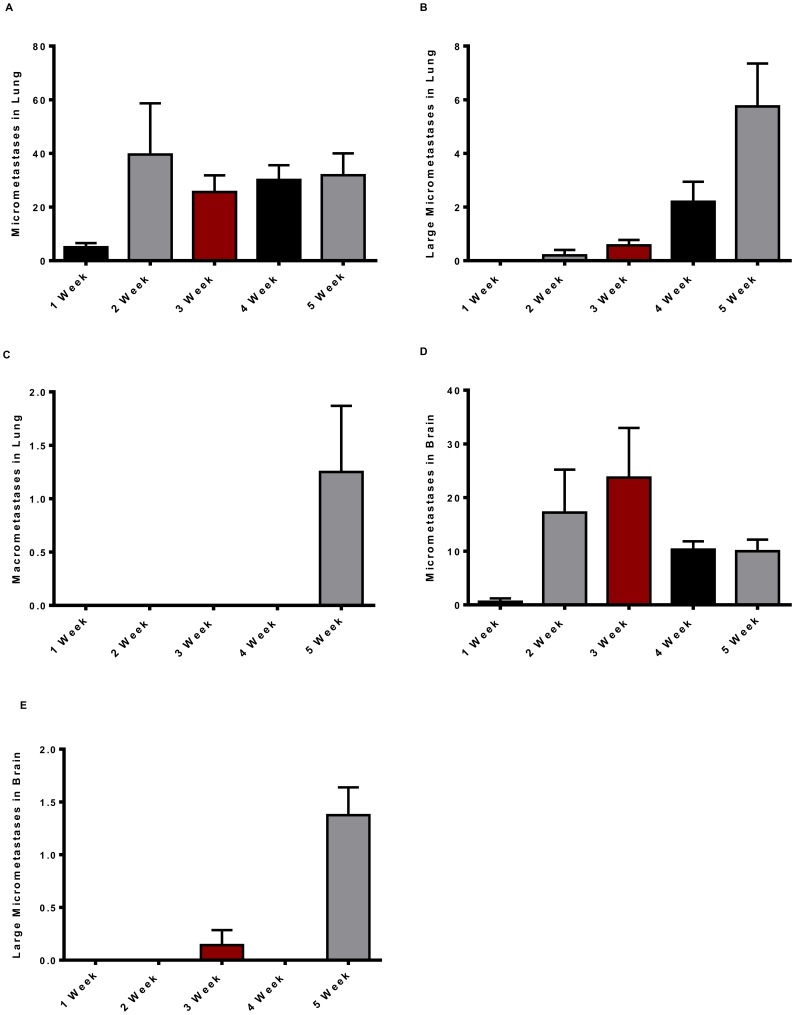
Development and quantification of lung and brain metastases: Timecourse. Micrometastases examination in lungs and brain of BALB/cJ mice implanted with 7,500 4T1-Luc2GFP cells into mammary fat pad. Lungs and brain excised, examined for metastases, degree of vascularity, and imaged at weeks two-five. (**A**) Lung micrometastases. (**B**) Lung large micrometastases. (**C**) Lung macrometastases. (**D**) Brain micrometastases. (**E**) Brain large micrometastases. All data are mean ± *SEM* from two independent experiments with 5–8 mice for experiment one and 7–11 mice for experiment two.

The progression of lung and brain metastases is illustrated in **figure 4**. Micrometastases were observed in the lung one week after implantation. Starting at week two, an average of 40 micrometastases per animal was observed in over 90% of animals each week through week six (**Fig. 4A**). Large lung micrometastases increased from weeks two to five both in terms of number (**Fig. 4C**) and frequency (10% of animals in week two, 58% in week three, 78% in week four and 80% in week five). Lung macrometastases were not observed until four weeks (14% of animals) and five weeks (67% of animals) after implantation (**Fig. 4C**). The brain yielded fewer micrometastases with only a single animal out of 60 animals containing micrometastases one week after implantation (**Fig. 4D**). Despite the decrease in overall micrometastases, metastatic progression in the brain was comparable to the observed progression in the lung with the number of small brain micrometastases increasing at weeks two and three (**Fig. 4D**). There were on average only one-three large micrometastases in the brain at week five and six (**Fig. 4E**) versus up to 12 large micrometastases in the lung (**Fig. 4B**), and no brain macrometastases were observed at any time point (data not shown). At five weeks post-implantation, five of 20 animals (25%) had small and one-two large micrometastases but no macrometastases in their bone (**Fig. S1** for representative image) and one animal out of 20 (5%) had small micrometastases in their liver, at a similar density to that observed at week two in the lung (data not shown). Neither micrometastases nor macrometastases were present in the spleen or kidney at any time point (data not shown).

**Figure 5:**
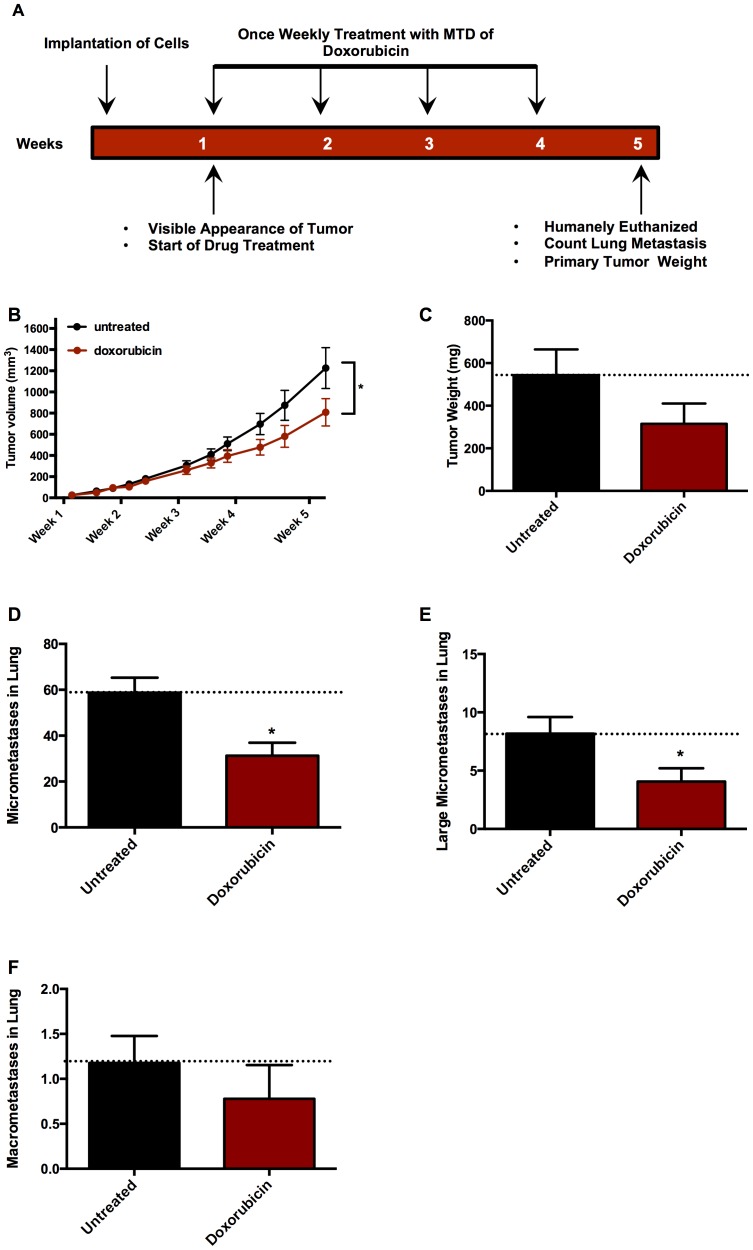
Efficacy of traditional chemotherapeutic agent in the 4T1 Luc2GPF mouse model. BALB/cJ mice implanted with 7,500 4T1-Luc2GFP cells were treated with the maximal tolerated dose (MTD) of doxorubicin, the dose shown not to result in any gross toxicity to the animal but shown to result in a small but insignificant loss in weight over the course of the experiment; or with carrier (5% Pharmasolve and 5% Solutol HS in saline), or with injectable saline as control once a week after visible primary tumor formation, for five weeks. (**A**) Flowchart indicating timing of cell implantation, doxorubicin treatments, and experimental determinations. (**B**) Tumors volume (mm^3^) over time measured by calipers. (**C**) End weight of tumors (mg), (**D**) Lung micrometastases. (**E**) Lung large micrometastases. (**F**) Lung macrometastases. Data representative of two independent experiments *n* = 15 mice for first experiment, and *n* = 10 mice for second experiment. Tumor volumes were calculated using the ellipsoidal method, volume (mm^3^) = 0.52 × length × width^2^. Data are mean ± *SEM* and a two way ANOVA/Tukey post-test was performed (*p<0.05 and **p<0.01).

Finally, the effect of a conventional anticancer chemotherapeutic agent, the cytotoxic anthracycline doxorubicin used in many patients with breast cancer, was examined with results as shown in **figure 5**. After five weeks of doxorubicin treatment (**Fig. 5A**), primary tumor volumes (**Fig. 5B**) were significantly reduced when compared to untreated (saline) -treated animals. Doxorubicin treatment also significantly diminished micrometastases (**Fig. 5D**) and large micrometastases (**Fig. 5E**), but not lung macrometastases **(** **Fig. 5F** **)** in the lung compared to untreated animals. All treatments were well tolerated with no differences in animal weight between groups throughout the study (data not shown).

## Discussion

In this work, a more natural model of lung metastasis progression was developed by optimizing a syngeneic TNBC mouse model. We show that by optimizing cell number, we can get over 90% primary tumor take within 10 days of implantation. We observe lung and brain micrometastasis beginning at one week and continuing to five weeks after implantation. We also see bone and liver micrometastases at week five, though the incidence of these is much less than in the lungs or brain. We also show that macrometastases are observed in the lungs of most animals at five weeks after implantation and by six weeks most animals were moribund from their tumor burden. This optimization scheme is shown in figure **1A**. Finally, we show that this model can be used to assess the effects of chemotherapeutic agents like doxorubicin on primary tumor growth and metastases and can delineate the steps in the metastatic process at which the agent is effective.

The optimization of the model identified two important technical factors as being critical for achieving successful tumor growth with far fewer cells than are typically used. First was how the cells are prepared and handled. We discovered that the cells need to be implanted within 20 minutes and should not be placed on ice (data not shown). Second, the number of cells needs to be optimized. Each investigator should probably determine this optimization, as it could depend on factors peculiar to each cell type (e.g. passage number) and laboratory. **Table 1** shows a review of the literature with respect to the 4T1 model being used for metastasis evaluation. The number of cells implanted ranged from 40,000 to 1 million; most cells were placed into a fat pad; in a few papers the primary tumor was removed at two weeks because it had become too large; metastases were evaluated from three-six weeks after implantation; and by far the lung was the major site of metastasis evaluation, though a few papers looked in other organs. Most papers evaluated just macrometastases, though two papers [17], [18] also qualitatively assessed micrometastases. From this literature search, it is clear that the modification of this model described in this work is the first to use as low a cell number and the first to evaluate metastatic progression over time.

In the initial cell titration experiment, it was found that primary tumor take was substantially lower (<50%) in animals implanted with 1,500 cells or less. This is likely due to the intact immune system of the BALB/cJ mice, rejecting the tumor cells or to the cells being insufficient in number to re-model the local extracellular matrix. It has been known for many years that lymphocytes can affect tumor growth [19] and it has been found that natural killer cells, tumor-associated macrophages (TAMs), T-regulatory (Treg) and dendritic cells play a role in tumorigenesis [20]–[22]. Further, stimulation of all of these immune cell types has been shown in response to tumor implantation in BALB/cJ mice, those used in our model [23]–[25]. An interesting finding was that decreasing cell number to 1,500 cells and below still maintained lung micrometastasis, but likely lengthened the time to macrometastasis formation, because animals implanted with lower numbers of cells rarely had macrometastases five weeks post implantation as compared to animals implanted with 7,500 cells. This finding also confirms that metastasis can occur very early, as has been previously reported [26]. The significance is that even though a primary tumor has been completely extricated by surgery, patients can have these “ticking time bombs” disseminated to other organs where they die out over time, remain dormant, or eventually activate and grow into a second generation, delayed metastasis that is inevitable refractory to treatment.

We used fluorescently labeled dextran as an *in* *vivo* blood vessel stain to detect the presence of a vasculature as a criterion for separating multicellular avascular micrometastases from vascular macrometastases [27]. From this staining, a notable finding was that the number of large micrometastases, but not small micrometastases, trended with the size of the primary tumor. This trend seems to go against the idea that a larger tumor is releasing more cells to distal sites such as the lung. It is possible that the lung reaches a maximal load, in this case around 60 micrometastases. It is also possible that many of the micrometastases die soon after seeding and are replaced by other cells [28]. A third alternative is that seeded cells enter a state of dormancy initiated by the normal lung extracellular matrix and other cellular factors [28], [29]. It is most reasonable to assume that the findings are a combination of all three cellular fate alternatives. The dormancy idea could be examined by removing the primary tumor in the first couple of weeks after implantation, thus effectively ablating the source of constant seeding of cells from the primary tumor. As others have shown, removing the primary tumor could also extend the time before animals become sick, enhance lung macrometastasis and potentially allow for a more thorough examination of macrometastases [14], [30]. An explanation as to why the number of large avascular micrometastases trends with primary tumor size could be something as simple as time - that is, that the small micrometastases are seeded early in the lung and have a similar growth pattern as the primary tumor. It is also possible that the primary tumor releases paracrine factors that stimulate the conversion of small to large micrometastases [31].

A compelling question in cancer research is: what triggers the activation of micrometastases to macrometastases? This is important because, as mentioned earlier, macrometastasis and/or local recurrence are most often what end up killing the cancer patient. Angiogenesis, or the so called angiogenic switch from an avascular to a vascular tumor, is necessary and also appears sufficient for macrometastasis activation [32]. A question though is whether the angiogenic switch is the proximal event for macrometastasis activation or is it simply occurring as a result of a more proximal event? Similar, driver mutations like activation of c-MYC are necessary and sufficient for macrometastasis activation [33] but these mutations are not observed in all tumor types, so can they explain mechanistically global macrometastasis activation? The 4T1 metastatic progression model described here can be used to study this reactivation process over time by isolating small and large micrometastases from the lung and by comparing these genetically, epigenetically and biochemically to activated macrometastases. While not completed in these studies, the 4T1 cells used also contain a luciferase gene, so more sensitive *in situ* imaging could be done to track the progression of single micrometastases over time.

Finally, this model can also be used for antimetastatic drug testing. Our studies showed that doxorubicin, a conventional cytotoxic agent still used as initial therapy of TNBC, resulted in decreased primary tumor growth and numbers of small and large micrometastases but had little effect on lung macrometastases. This finding is in keeping with clinical findings that doxorubicin and indeed most cytotoxic chemotherapeutic agents work well on primary tumors but not on metastatic disease [3], [34].

Taken together, we have developed a technically simple physiologically relevant breast to lung/brain metastatic progression model in immune proficient mice. This model can be used to study temporal aspects of the process of metastasis, to test anti-metastatic agents and to further define relevant molecular targets to intervene therapeutically in this critical process.

## Supporting Information

Figure S1 **Micrometastases in murine bone.** BALB/cJ female mice (*n* = 20) were implanted with 7,500 dtTomato red-labeled 4T1 cells. Five weeks after implantation animals were euthanized, tibia and femur bones were cleaned, and fluorescence was assessed. (**A**) Tibia with micrometastatic cells (red) magnification x 20. (**B**) Insert depicting magnification x 200 with white arrows indicating micrometastatic cells.
(TIF)

## References

[1] Weigelt B, Peterse JL, van’t Veer LJ (2005) Breast cancer metastasis: markers and models. Nat Rev Cancer 5: 591–602 10.1038/nrc1670 16056258

[2] Nkrumah FK, Perkins IV (1976) Relapse in Burkitt’s lymphoma. Int J Cancer 17: 455–460. 1279038 10.1002/ijc.2910170407

[3] Yuhas JM, Toya RE, Wagner E (1975) Specific and nonspecific stimulation of resistance to the growth and metastasis of the line 1 lung carcinoma. Cancer Res 35: 242–244. 1109793

[4] Clayton F, Hopkins CL (1993) Pathologic correlates of prognosis in lymph node-positive breast carcinomas. Cancer 71: 1780–1790. 8383579 10.1002/1097-0142(19930301)71:5<1780::aid-cncr2820710512>3.0.co;2-2

[5] Naumov GN, Townson JL, MacDonald IC, Wilson SM, Bramwell VHC, et al (2003) Ineffectiveness of doxorubicin treatment on solitary dormant mammary carcinoma cells or late-developing metastases. Breast Cancer Res Treat 82: 199–206 10.1023/B:BREA.0000004377.12288.3c 14703067

[6] Mittempergher L, Saghatchian M, Wolf DM, Michiels S, Canisius S, et al. (2013) A gene signature for late distant metastasis in breast cancer identifies a potential mechanism of late recurrences. Molecular Oncology: 1–13. doi:10.1016/j.molonc.2013.07.006. PMC5528450 23910573

[7] Nishikawa S, Dewi DL, Ishii H, Konno M, Haraguchi N, et al (2012) Transcriptomic study of dormant gastrointestinal cancer stem cells. Int J Oncol 41: 979–984 10.3892/ijo.2012.1531 22735680

[8] Francia G, Cruz-Munoz W, Man S, Xu P, Kerbel RS (2011) Mouse models of advanced spontaneous metastasis for experimental therapeutics. Nat Rev Cancer 11: 135–141 10.1038/nrc3001 21258397 PMC4540342

[9] Viney JL (1995) Transgenic and gene knockout mice in cancer research. Cancer Metastasis Rev 14: 77–90. 7554032 10.1007/BF00665792

[10] Grippo PJ, Sandgren EP (2000) Highly invasive transitional cell carcinoma of the bladder in a simian virus 40 T-antigen transgenic mouse model. The American Journal of Pathology 157: 805–813 10.1016/S0002-9440(10)64594-4 10980120 PMC1885716

[11] Gingrich JR, Barrios RJ, Morton RA, Boyce BF, DeMayo FJ, et al (1996) Metastatic prostate cancer in a transgenic mouse. Cancer Res 56: 4096–4102. 8797572

[12] Webster MA, Muller WJ (1994) Mammary tumorigenesis and metastasis in transgenic mice. Semin Cancer Biol 5: 69–76. 8186390

[13] Hanahan D, Weinberg RA (2011) Hallmarks of Cancer: The Next Generation. Cell 144: 646–674 10.1016/j.cell.2011.02.013 21376230

[14] Pulaski BA, Ostrand-Rosenberg S (2001) Mouse 4T1 breast tumor model. Curr Protoc Immunol Chapter 20: Unit20.2 10.1002/0471142735.im2002s39 18432775

[15] Ihnat MA, Nervi AM, Anthony SP, Kaltreider RC, Warren AJ, et al (1999) Effects of mitomycin C and carboplatin pretreatment on multidrug resistance-associated P-glycoprotein expression and on subsequent suppression of tumor growth by doxorubicin and paclitaxel in human metastatic breast cancer xenografted nude mice. Oncol Res 11: 303–310. 10757444

[16] Chow KS, Jun D, Helm KM, Wagner DH, Majka SM (2011) Isolation & characterization of Hoechst(low) CD45(negative) mouse lung mesenchymal stem cells. J Vis Exp: e3159. doi:10.3791/3159. PMC3227187 22064472

[17] Erin N, Boyer PJ, Bonneau RH, Clawson GA, Welch DR (2004) Capsaicin-mediated denervation of sensory neurons promotes mammary tumor metastasis to lung and heart. Anticancer Res 24: 1003–1009. 15161056

[18] Wenzel J, Zeisig R, Fichtner I (2010) Inhibition of metastasis in a murine 4T1 breast cancer model by liposomes preventing tumor cell-platelet interactions. Clin Exp Metastasis 27: 25–34 10.1007/s10585-009-9299-y 19916050

[19] Burnet M (1957) Cancer; a biological approach. I. The processes of control. Br Med J 1: 779–786. 13404306 10.1136/bmj.1.5022.779 PMC1973174

[20] Toh B, Chew V, Dai X, Khoo K, Tham M, et al (2012) Immune predictors of cancer progression. Immunol Res 53: 229–234 10.1007/s12026-012-8288-4 22407576

[21] Hao N-B, Lü M-H, Fan Y-H, Cao Y-L, Zhang Z-R, et al (2012) Macrophages in tumor microenvironments and the progression of tumors. Clin Dev Immunol 2012: 948098 10.1155/2012/948098 22778768 PMC3385963

[22] Marabelle A, Kohrt H, Sagiv-Barfi I, Ajami B, Axtell RC, et al (2013) Depleting tumor-specific Tregs at a single site eradicates disseminated tumors. J Clin Invest 123: 2447–2463 10.1172/JCI64859DS1 23728179 PMC3668834

[23] Mahoney KH, Miller BE, Heppner GH (1985) FACS quantitation of leucine aminopeptidase and acid phosphatase on tumor-associated macrophages from metastatic and nonmetastatic mouse mammary tumors. J Leukoc Biol 38: 573–585. 2413152 10.1002/jlb.38.5.573

[24] Sawant A, Hensel JA, Chanda D, Harris BA, Siegal GP, et al (2012) Depletion of plasmacytoid dendritic cells inhibits tumor growth and prevents bone metastasis of breast cancer cells. J Immunol 189: 4258–4265 10.4049/jimmunol.1101855 23018462 PMC3531993

[25] Pollack VA, Fidler IJ (1982) Use of young nude mice for selection of subpopulations of cells with increased metastatic potential from nonsyngeneic neoplasms. J Natl Cancer Inst 69: 137–141. 6954306

[26] Melchior SW, Corey E, Ellis WJ, Ross AA, Layton TJ, et al (1997) Early tumor cell dissemination in patients with clinically localized carcinoma of the prostate. Clin Cancer Res 3: 249–256. 9815680

[27] D’Amato R, Wesolowski E, Smith LE (1993) Microscopic visualization of the retina by angiography with high-molecular-weight fluorescein-labeled dextrans in the mouse. Microvasc Res 46: 135–142 10.1006/mvre.1993.1042 7504160

[28] Luzzi KJ, MacDonald IC, Schmidt EE, Kerkvliet N, Morris VL, et al (1998) Multistep nature of metastatic inefficiency: dormancy of solitary cells after successful extravasation and limited survival of early micrometastases. The American Journal of Pathology 153: 865–873 10.1016/S0002-9440(10)65628-3 9736035 PMC1853000

[29] Almog N (2006) Prolonged dormancy of human liposarcoma is associated with impaired tumor angiogenesis. The FASEB Journal 20: 947–949 10.1096/fj.053946fje 16638967

[30] Al-Sahaf O, Wang JH, Browne TJ, Cotter TG, Redmond HP (2010) Surgical injury enhances the expression of genes that mediate breast cancer metastasis to the lung. Ann Surg 252: 1037–1043 10.1097/SLA.0b013e3181efc635 21107114

[31] Barkan D, Touny El LH, Michalowski AM, Smith JA, Chu I, et al (2010) Metastatic Growth from Dormant Cells Induced by a Col-I-Enriched Fibrotic Environment. Cancer Res 70: 5706–5716 10.1158/0008-5472.CAN-09-2356 20570886 PMC3436125

[32] Gimbrone MA, Leapman SB, Cotran RS, Folkman J (1972) Tumor dormancy *in* *vivo* by prevention of neovascularization. J Exp Med 136: 261–276. 5043412 10.1084/jem.136.2.261 PMC2139203

[33] Shachaf CM, Kopelman AM, Arvanitis C, Karlsson A, Beer S, et al (2004) MYC inactivation uncovers pluripotent differentiation and tumour dormancy in hepatocellular cancer. Nature 431: 1112–1117 10.1038/nature03043 15475948

[34] Heppner GH, Dexter DL, DeNucci T, Miller FR, Calabresi P (1978) Heterogeneity in drug sensitivity among tumor cell subpopulations of a single mammary tumor. Cancer Res 38: 3758–3763. 698935

[35] Aslakson CJ, Miller FR (1992) Selective events in the metastatic process defined by analysis of the sequential dissemination of subpopulations of a mouse mammary tumor. Cancer Res 52: 1399–1405. 1540948

[36] Pulaski BA, Ostrand-Rosenberg S (1998) Reduction of established spontaneous mammary carcinoma metastases following immunotherapy with major histocompatibility complex class II and B7.1 cell-based tumor vaccines. Cancer Res 58: 1486–1493. 9537252

[37] Lelekakis M, Moseley JM, Martin TJ, Hards D, Williams E, et al (1999) A novel orthotopic model of breast cancer metastasis to bone. Clin Exp Metastasis 17: 163–170. 10411109 10.1023/a:1006689719505

[38] Muraoka RS, Dumont N, Ritter CA, Dugger TC, Brantley DM, et al (2002) Blockade of TGF-beta inhibits mammary tumor cell viability, migration, and metastases. J Clin Invest 109: 1551–1559 10.1172/JCI15234 12070302 PMC151012

[39] Connolly EM, Harmey JH, O’Grady T, Foley D, Roche-Nagle G, et al (2002) Cyclo-oxygenase inhibition reduces tumour growth and metastasis in an orthotopic model of breast cancer. British Journal of Cancer 87: 231–237 10.1038/sj.bjc.6600462 12107848 PMC2376100

[40] Yang J, Mani SA, Donaher JL, Ramaswamy S, Itzykson RA, et al (2004) Twist, a master regulator of morphogenesis, plays an essential role in tumor metastasis. Cell 117: 927–939 10.1016/j.cell.2004.06.006 15210113

[41] Hiraga T, Williams PJ, Ueda A, Tamura D, Yoneda T (2004) Zoledronic acid inhibits visceral metastases in the 4T1/luc mouse breast cancer model. Clin Cancer Res 10: 4559–4567 10.1158/1078-0432.CCR-03-0325 15240548

[42] Smith MCP, Luker KE, Garbow JR, Prior JL, Jackson E, et al (2004) CXCR4 regulates growth of both primary and metastatic breast cancer. Cancer Res 64: 8604–8612 10.1158/0008-5472.CAN-04-1844 15574767

[43] Bausero MA, Page DT, Osinaga E, Asea A (2004) Surface expression of Hsp25 and Hsp72 differentially regulates tumor growth and metastasis. Tumour Biol 25: 243–251 10.1159/000081387 15627887 PMC1764489

[44] Lirdprapamongkol K, Sakurai H, Kawasaki N, Choo M-K, Saitoh Y, et al (2005) Vanillin suppresses in vitro invasion and *in* *vivo* metastasis of mouse breast cancer cells. Eur J Pharm Sci 25: 57–65 10.1016/j.ejps.2005.01.015 15854801

[45] Samant RS, Debies MT, Hurst DR, Moore BP, Shevde LA, et al (2006) Suppression of murine mammary carcinoma metastasis by the murine ortholog of breast cancer metastasis suppressor 1 (Brms1). Cancer Lett 235: 260–265 10.1016/j.canlet.2005.04.032 15978719

[46] Nam J-S, Suchar AM, Kang M-J, Stuelten CH, Tang B, et al (2006) Bone sialoprotein mediates the tumor cell-targeted prometastatic activity of transforming growth factor beta in a mouse model of breast cancer. Cancer Res 66: 6327–6335 10.1158/0008-5472.CAN-06-0068 16778210 PMC1528715

[47] Heimburg J, Yan J, Morey S, Glinskii OV, Huxley VH, et al (2006) Inhibition of spontaneous breast cancer metastasis by anti-Thomsen-Friedenreich antigen monoclonal antibody JAA-F11. Neoplasia 8: 939–948 10.1593/neo.06493 17132226 PMC1716011

[48] Li H, Dutuor A, Fu X, Zhang X (2007) Induction of strong antitumor immunity by an HSV-2-based oncolytic virus in a murine mammary tumor model. J Gene Med 9: 161–169 10.1002/jgm.1005 17266169

[49] Tao K, Fang M, Alroy J, Sahagian GG (2008) Imagable 4T1 model for the study of late stage breast cancer. BMC Cancer 8: 228 10.1186/1471-2407-8-228 18691423 PMC2529338

[50] Roy Das L, Pathangey LB, Tinder TL, Schettini JL, Gruber HE, et al (2009) Breast-cancer-associated metastasis is significantly increased in a model of autoimmune arthritis. Breast Cancer Res 11: R56 10.1186/bcr2345 19643025 PMC2750117

[51] Ma L, Reinhardt F, Pan E, Soutschek J, Bhat B, et al (2010) Therapeutic silencing of miR-10b inhibits metastasis in a mouse mammary tumor model. Nat Biotechnol 28: 341–347 10.1038/nbt.1618 20351690 PMC2852471

[52] Kim EJ, Choi M-R, Park H, Kim M, Hong JE, et al (2011) Dietary fat increases solid tumor growth and metastasis of 4T1 murine mammary carcinoma cells and mortality in obesity-resistant BALB/c mice. Breast Cancer Res 13: R78 10.1186/bcr2927 21834963 PMC3236342

