# Supplementary figures and images for: Development and Characterization of a Preclinical Model of Breast Cancer Lung Micrometastatic to Macrometastatic Progression
Source: PLoS One. 2014 May 30;9(5):e98624. doi: 10.1371/journal.pone.0098624 (PMC4039511; doi:10.1371/journal.pone.0098624)

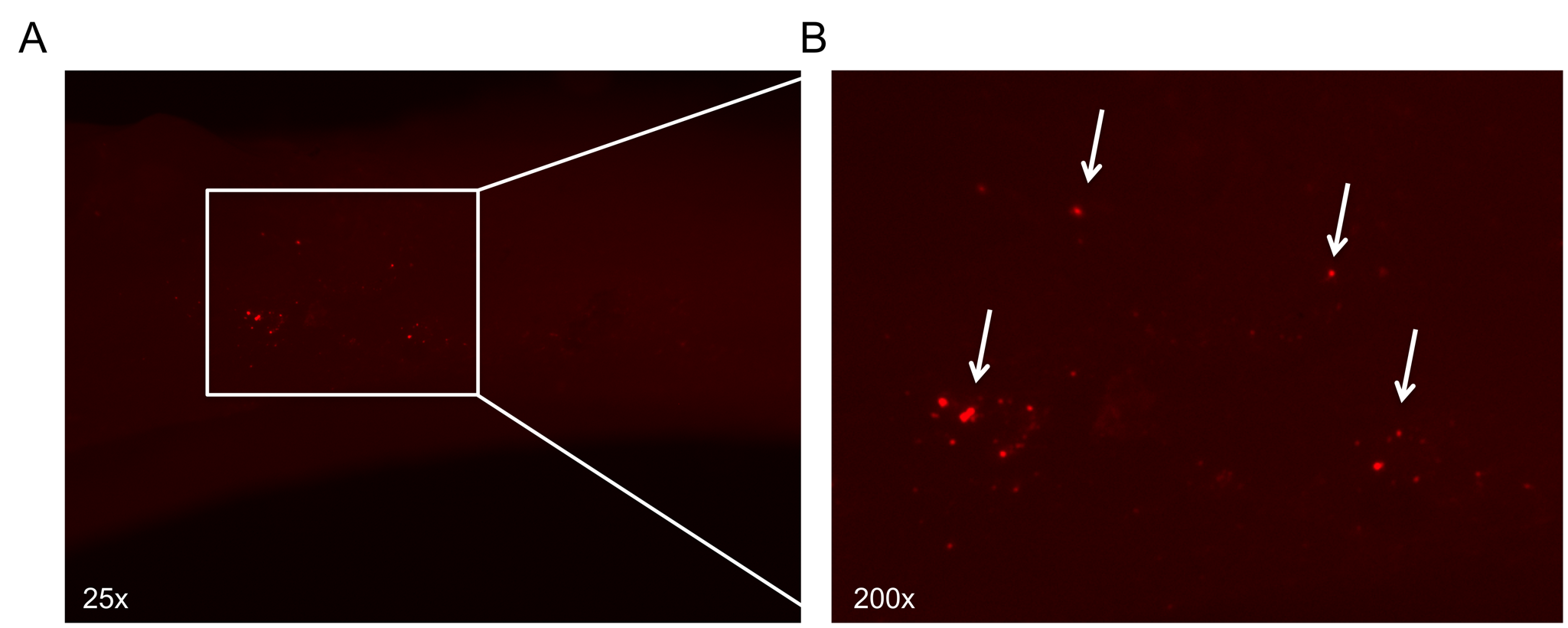

Supplement: Figure S1 — Micrometastases in murine bone. BALB/cJ female mice (n = 20) were implanted with 7,500 dtTomato red-labeled 4T1 cells. Five weeks after implantation animals were euthanized, tibia and femur bones were cleaned, and fluorescence was assessed. (A) Tibia with micrometastatic cells (red) magnification x 20. (B) Insert depicting magnification x 200 with white arrows indicating micrometastatic cells. (TIF) [file pone.0098624.s001.tif]
